# Increased Expression of Cytotoxic T-Lymphocyte−Associated Protein 4 by T Cells, Induced by B7 in Sera, Reduces Adaptive Immunity in Patients With Acute Liver Failure

**DOI:** 10.1053/j.gastro.2017.03.023

**Published:** 2017-07

**Authors:** Wafa Khamri, Robin D. Abeles, Tie Zheng Hou, Amy E. Anderson, Ahmed El-Masry, Evangelos Triantafyllou, Christine Bernsmeier, Fin S. Larsen, Arjuna Singanayagam, Nobuaki Kudo, Lucia A. Possamai, Fanny Lebosse, Georg Auzinger, William Bernal, Christopher Willars, Christopher J. Weston, Giovanna Lombardi, Julia Wendon, Mark Thursz, Charalambos G. Antoniades

**Affiliations:** 1 Division of Digestive Diseases, Imperial College London, United Kingdom; 2 Institute of Liver Studies, King's College London, United Kingdom; 3 Institute of Immunity and Transplantation, University College London, United Kingdom; 4 Institute of Cellular Medicine, Newcastle University, Newcastle, United Kingdom; 5 Department of Hepatology, Rigshospitalet, Copenhagen, Denmark; 6 Institute of Reproductive and Developmental Biology, Imperial College London, United Kingdom; 7 Centre for Liver Research and National Institute for Health Research, Biomedical Research Unit, University of Birmingham, United Kingdom; 8 Medical Research Council Centre for Transplantation, King's College London, United Kingdom

**Keywords:** Immune Regulation, Liver Disease, Treatment, Infection Susceptibility, AALF, acetaminophen-induced acute liver failure, ALF, acute liver failure, APAP, acetaminophen, CLD, chronic liver disease, CTLA4, cytotoxic T-lymphocyte−associated molecule-4, DC, dendritic cell, HC, healthy control, HSEC, hepatic sinusoidal endothelial cell, IFN, interferon, IL, interleukin, IQR, interquartile range, PE, plasma exchange, sB7, soluble B7

## Abstract

**Background & Aims:**

Patients with acute liver failure (ALF) have defects in innate immune responses to microbes (immune paresis) and are susceptible to sepsis. Cytotoxic T-lymphocyte−associated protein 4 (CTLA4), which interacts with the membrane receptor B7 (also called CD80 and CD86), is a negative regulator of T-cell activation. We collected T cells from patients with ALF and investigated whether inhibitory signals down-regulate adaptive immune responses in patients with ALF.

**Methods:**

We collected peripheral blood mononuclear cells from patients with ALF and controls from September 2013 through September 2015 (45 patients with ALF, 20 patients with acute-on-chronic liver failure, 15 patients with cirrhosis with no evidence of acute decompensation, 20 patients with septic shock but no cirrhosis or liver disease, and 20 healthy individuals). Circulating CD4^+^ T cells were isolated and analyzed by flow cytometry. CD4^+^ T cells were incubated with antigen, or agonist to CD3 and dendritic cells, with or without antibody against CTLA4; T-cell proliferation and protein expression were quantified. We measured levels of soluble B7 molecules in supernatants of isolated primary hepatocytes, hepatic sinusoidal endothelial cells, and biliary epithelial cells from healthy or diseased liver tissues. We also measured levels of soluble B7 serum samples from patients and controls, and mice with acetaminophen-induced liver injury using enzyme-linked immunosorbent assays.

**Results:**

Peripheral blood samples from patients with ALF had a higher proportion of CD4^+^ CTLA4^+^ T cells than controls; patients with infections had the highest proportions. CD4^+^ T cells from patients with ALF had a reduced proliferative response to antigen or CD3 stimulation compared to cells from controls; incubation of CD4^+^ T cells from patients with ALF with an antibody against CTLA4 increased their proliferative response to antigen and to CD3 stimulation, to the same levels as cells from controls. CD4^+^ T cells from controls up-regulated expression of CTLA4 after 24−48 hours culture with sera from patients with ALF; these sera were found to have increased concentrations of soluble B7 compared to sera from controls. Necrotic human primary hepatocytes exposed to acetaminophen, but not hepatic sinusoidal endothelial cells and biliary epithelial cells from patients with ALF, secreted high levels of soluble B7. Sera from mice with acetaminophen-induced liver injury contained high levels of soluble B7 compared to sera from mice without liver injury. Plasma exchange reduced circulating levels of soluble B7 in patients with ALF and expression of CTLA4 on T cells.

**Conclusions:**

Peripheral CD4^+^ T cells from patients with ALF have increased expression of CTLA4 compared to individuals without ALF; these cells have a reduced response to antigen and CD3 stimulation. We found sera of patients with ALF and from mice with liver injury to have high concentrations of soluble B7, which up-regulates CTLA4 expression by T cells and reduces their response to antigen. Plasma exchange reduces levels of B7 in sera from patients with ALF and might be used to restore antimicrobial responses to patients.

**Editor's Notes**

**Background and Context**

Systemic innate immune defects are well-characterized as contributors to immuneparesis and susceptibility to infections in patients with acute liver failure (ALF). However, dysfunctions in adaptive immune responses were unexplored.

**New Findings**

Following acute liver injury, peripheral CD4^+^ T cells in ALF patients display an increased expression of cytotoxic T lymphocyte-associated molecule-4 (CTLA4) with an inhibitory functional aspect that dampens protective immunity.

**Limitations**

Experimental models for assessing the role of CTLA4 in ALF.

**Impact**

This work has identified a novel therapeutic target to reverse immune dysfunctions in patients with ALF.

Acute liver failure (ALF) occurs after a severe hepatic insult resulting in a rapidly progressive clinical syndrome characterized by jaundice, encephalopathy, coagulopathy, and multiple organ dysfunction.^1^, ^2^ Although the initiating event in ALF is acute hepatocellular death, mortality is attributable to a profound activation of systemic inflammatory response syndrome and multiple organ dysfunction.^1^, ^2^, ^3^ Recent studies identify defects in innate immune responses to microbial cues, termed *immune paresis*, which cause an increased susceptibility to secondary infections, a leading cause of mortality in ALF.^4^, ^5^, ^6^, ^7^

After acute tissue injury, intracellular components, defined as damage-associated molecular pattern molecules or alarmins, are released by necrotic and apoptotic cells and act as “danger” signal molecules that trigger organ-specific and systemic inflammatory response syndrome.^8^, ^9^ Studies identify profound elevations in these danger signals in patients with acute hepatic inflammatory disorders, which have recently been shown to modulate the function of myeloid and possibly lymphoid cells.^10^, ^11^, ^12^

Substantial evidence exists in nonhepatic inflammatory disorders (eg, severe trauma, pancreatitis, septic shock) of suppression in T-cell−mediated antimicrobial responses that account for the immune paresis and infectious complications encountered in these patients.^9^, ^13^ The acquired immune dysfunction reported in sepsis includes increased regulatory T cells with elevated levels of inhibitory receptor including PD-1 and CTLA4,^13^, ^14^ which correlate with reduced interferon (IFN) gamma production and low T-cell proliferative capacity.^15^ In murine models of sepsis, CTLA4 was also reported to be elevated on CD4^+^ and CD8^+^ T cells.^16^ Accordingly, blocking PD-1 and CTLA4-mediated negative regulatory pathways demonstrated improved survival in animal models of bacterial and fungal sepsis, increased pathogen clearance, and reversed T-cell dysfunction in patients with sepsis.^16^, ^17^ However, it remains to be determined whether defects in peripheral adaptive immune responses play a role in the immune paresis reported in ALF.

CTLA4 (CD152) is a well-characterized negative regulator expressed on T cells that binds to the same ligands (CD80 and CD86) as CD28, but with a higher affinity.^18^ Upon T-cell activation, CTLA4 cycles to the cell surface and exerts its inhibitory effect, which results in attenuated T-cell responses and inhibition of interleukin (IL)-2 secretion, a critical cytokine for T-cell expansion.^19^, ^20^ In humans, CTLA4 has been implicated in numerous autoimmune diseases, such as rheumatoid arthritis and systemic lupus erythematosus.^21^, ^22^ In severe sepsis, CTLA4 is up-regulated, resulting in impairment in T-cell activation and antimicrobial responses.^17^, ^23^ CTLA4 was the first immune checkpoint receptor to be clinically targeted and several studies have demonstrated that antibody blockade of CTLA4 could result in anti-tumor immunity.^24^, ^25^

In this study, we sought to assess the immunologic competence of CD4^+^ T cells in patients with ALF by examining their phenotype and function. Here, we identify adaptive immune dysfunction in CD4^+^ T cells due to a sustained expression of the negative regulator of T-cell activation, CTLA4, via mechanisms that involve soluble B7 (sB7) ligands. The impairment of peripheral CD4^+^ T cells adaptive responses in ALF can contribute to the susceptibility to infections seen in these patients.

## Methods

### Patient Characteristics

All patients were consecutively recruited from September 2013 to September 2015. Subjects were recruited to the study within 24 hours of admission to the liver intensive therapy unit or liver wards. Patients were categorized into the following groups: ALF (n = 45: 35 acetaminophen-induced ALF [AALF] and 10 non-AALF, patients with acute-on-chronic liver failure (ACLF; n = 20), chronic liver disease (CLD) patients with cirrhosis with no evidence of acute decompensation (n = 15), patients with septic shock with no underlying cirrhosis/liver disease (sepsis group; n = 20), healthy controls (HC; n = 20). A subset of ALF patients underwent sequential sampling on day 3 (n = 7), day 7 (n = 8), and day 14 (n = 5) after admission. In addition, a subset of ACLF patients underwent sequential sampling on day 3 (n = 5), day 7 (n = 4), and day 14 (n = 5). When plasma exchange (PE) was instituted in ALF patients recruited, additional sampling was performed immediately before PE (pre-PE, n = 7) and within 8 hours after completion of PE (post-PE; n = 7). All patients who fulfilled criteria for ALF were transferred to liver intensive therapy unit based on published referral guidelines for both AALF and non-AALF.^26^ Patients with ACLF fulfilled the established diagnostic criteria developed by the European Association for the Study of the Liver-Chronic Liver Failure consortium.^27^ This study was approved by the King’s College Hospital ethics committee (12/LO/0167). Informed consent was obtained by the next of kin if patients were not able unable to provide consent.

### Phenotyping Using Flow Cytometry

Peripheral blood mononuclear cells isolated through density-gradient centrifugation were surface stained for CD3, CD4, CD8, CD45RA, CD45RO, CCR-7, CTLA4, PD-1, CD25, CD62L, CD28, CD40L, and CD127. Twelve-color flow cytometric analyses were performed using an LSR Fortessa flow cytometer and data were acquired using BD FACSDiva software (Becton Dickinson Ltd, Oxford, UK).

### Antigen Recall Responses and CD4^+^ T-Cell Proliferation

Specific CD4^+^ T-cell antigen-recall responses to a pool of HLA class II-restricted T-cell epitopes at 10 μg/mL (CTL Europe GmbH, Bonn, Germany) were examined in vitro for T-cell proliferation using either carboxyfluorescein succinimidyl ester or cell proliferation dye eFluor 670 (eBioscience, Hatfield, UK) labeling, in the presence or absence of anti-CTLA4 blocking antibody (10 μg/mL; eBioscience). All blocking antibodies used in this study were referenced neutralizing antibodies from the manufacturers. CD4^+^ T-cell proliferation was tested by labeling peripheral blood mononuclear cells with carboxyfluorescein succinimidyl ester before being placed in culture for 6 days. Peripheral blood mononuclear cells were cultured in RPMI 1640 (Thermo Fisher Scientific, Hemel Hempstead, UK) supplemented with 10% human AB serum (PAA Laboratories Ltd, West Yorkshire, UK). After stimulation for 6 days at 37°C in 5% CO_2_, cells were harvested and stained for surface markers (CD3, CD4, and CD8). Data were collected using LSR Fortessa (Becton Dickinson) and analyzed using FlowLogic (Inivai Technologies Pty Ltd, Victoria, Australia).

### Dendritic Cell CD4^+^ T-Cell Co-Culture

Allogeneic dendritic cells (DCs) were generated as described previously.^28^ They were then co-cultured with CD4^+^ T cells from ALF patients in the presence of anti-CD3 (α-CD3) antibody (0.5 μg/mL) (eBioscience, Hatfield, UK) at optimal ratio of 1:40 (DC to CD4^+^ T cells) ratio, which was selected after a dose−response testing (ranging from 1:1 to 1:80 DC to CD4^+^ T cells ratio). Cells were co-cultured for 5 days in the presence or absence of anti-CTLA4 (α-CTLA4) (10 μg/mL). Cell division of CD4^+^ T cell was measured by the dilution of carboxyfluorescein succinimidyl ester dye using flow cytometry.

### Effect of Circulating Soluble B7 on Cytotoxic T-Lymphocyte−Associated Protein 4 Expression

CD4^+^ T cells isolated from HC were cultured in fresh medium for 24 and 48 hours at 5 × 10^5^ cells per well in the presence of 10% ALF (n = 14) or HC (n = 12) sera and screened for CTLA4 surface expression using flow cytometry. Supernatant were collected for subsequent detection of cytokines. Sera samples from both HC and ALF patients were pre-incubated with anti-human CD80 or CD86 neutralizing antibodies (α-CD80 or α-CD86) (R&D Systems, Abingdon, UK) for 45 minutes at room temperature before addition to CD4^+^ T cells isolated from healthy donors. Cell culture supernatants were collected for assessing cytokine secretion. Cells were harvested for phenotyping. Unless otherwise stated, sB7 molecules refer to sCD80 and sCD86.

### Primary Human Hepatocytes and Kupffer Cells

Cryopreserved hepatocytes (Invivogen, Paisley, UK) were cultured for 24 hours in the presence or absence of 20 mM acetaminophen (APAP) (Sigma, Dorset, UK). Cells were stained using apoptotic/necrotic cell kit (PromoCell GmbH, Heidelberg, Germany). Apoptosis and necrosis were detected using Annexin V, ethidium homodimer III, respectively, according to manufacturer’s instructions. Kupffer cells (Invivogen) were stimulated overnight in the presence of lipopolysaccharide (100 ng/mL). Supernatants from hepatocyte and Kupffer cell cultures were collected for assessment of sB7 molecules.

### Isolation and Culture of Hepatic Sinusoidal Endothelial Cells and Biliary Epithelial Cells

Hepatic sinusoidal endothelial cells (HSECs) and biliary epithelial cells were isolated according to methods described previously,^29^ approved by the University of Birmingham ethics committee (06/Q2702/61). HSECs were cultured in the presence of sera from ALF (n = 6) and HC (n = 6) at 25%. Supernatants were then collected and assessed for sB7 molecules. Sera preconditioned HSECs were cultured for an additional 24 hours in the presence of 10 ng/mL recombinant human tumor necrosis factor−α and 10 ng/mL IFN gamma (PeproTech, London, UK). Supernatants were collected for assessment of sB7 molecules.

### Co-Culture of Monocytes With Apoptotic Neutrophils

Neutrophils were isolated by density gradient centrifugation as described previously.^30^ Neutrophils were resuspended (10^6^ cells/mL) in fresh complete medium and incubated for 20 hours (37°C in 5% CO_2_) in 24-well plates (Corning Inc, Tewksbury, MA). Annexin-V kit (BD Biosciences) was used to determine the percentage of apoptotic neutrophils, which was >65%, as described previously.^30^ Next, CD14^+^ monocytes were resuspended (0.5 × 10^6^ cells/mL) and co-incubated in 1:2 ratio for 4 hours with apoptotic neutrophils. Supernatants were collected in order to measure sB7 levels by enzyme-linked immunosorbent assay.

### Enzyme-Linked Immunosorbent Assay and Meso Scale Discovery Multiplex Cytokine Detection System

Soluble B7 were assessed in cell culture supernatants, human sera, and APAP-induced liver injury model murine models using enzyme-linked immunosorbent assays. Cytokines were also assessed in cell culture supernatant using enzyme-linked immunosorbent assay and Multiplex Cytokine Detection System (Meso Scale Discovery, Gaithersburg, MD).

### Statistical Analysis

Parametric statistical analysis was performed using the Student *t* test. Nonparametric analysis was carried out using the Mann−Whitney *U* test, Wilcoxon matched-pairs signed rank and Kruskal−Wallis tests, and data are expressed as median (interquartile range [IQR]). For correlations of CD4^+^CTLA4^+^ T-cell frequency and clinical characteristics as well as correlations of sB7 ligands and disease severity indices, Spearman rank correlation coefficients were used. Statistical significance was assumed for *P* < .05. All analyses were performed using GraphPad Prism software (GraphPad Inc, La Jolla, CA).

Other details and additional experimental procedures are provided in the Supplementary Material.

## Results

### Patient Characteristics

There was no significant difference in median ages of ALF patients when compared to HC, while pathologic patients groups were significantly older (Supplementary Table 1). ALF patients have significantly higher biochemical and physiologic indices of acute liver injury (eg, Model for End-Stage Liver Disease, international normalized ratio, creatinine, and bilirubin) compared to CLD, ACLF, and sepsis patients (Supplementary Table 1). The number of circulating lymphocytes was reduced significantly in ALF patients when compared to CLD and ALCF patients (Supplementary Table 1), although no differences were seen when compared with sepsis patients. In addition, lymphocyte counts in AALF correlated negatively with indices of severity of liver injury (international normalized ratio: *r* = −0.285, *P* = .04; aspartate aminotransferase: *r* = −0.465, *P* = .001; systemic inflammatory response syndrome scores: *r* = −0.391, *P* = .009; and Model for End-Stage Liver Disease scores: *r* = −0.557, *P* = .0001). The demographic and key clinical characteristics of patients are summarized in Supplementary Table 1. Clinical data for ALF patients who underwent PE are summarized in Supplementary Table 2.

### Increased Frequency of Circulating CD4^+^ T Cells in Acute Liver Failure Patients With a Predominantly Naïve Phenotype

**Figure 1:**
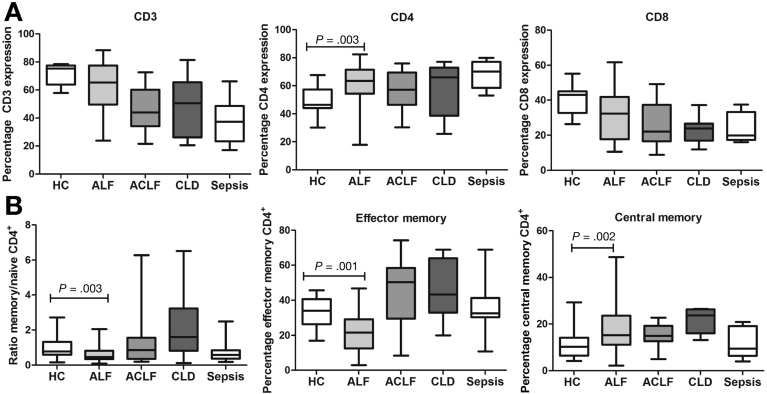
Phenotypic characterization of T-cell subsets in peripheral blood mononuclear cells of ALF patients. Gating strategy to define lymphocyte subsets is described in the Supplementary Materials (Supplementary Figure 1). (*A*) Immunophenotyping of circulating lymphocytes using flow cytometry. Data show the percentages of circulating CD3^+^ CD4^+^, and CD8^+^ T cells in ALF compared with HC and pathologic control groups. (*B*) Distribution of naïve CD45RO^−^RA^+^ and memory CD45RO^+^RA^−^ subpopulations within the expanded CD4^+^ T-cell population in ALF compared with healthy and pathologic control groups. *Left*: Data show ratios of percentage of expression. CD4^+^ T cells were subgrouped into effector memory T cell (CD3^+^CD4^+^CD8^−^CCR7^+^CD45RO^−^) (*middle panel*) and T_cm_ (CD3^+^CD4^+^CD8^−^CCR7^+^CD45RO^+^) populations (*right panel*). Percentages of expression are shown (HC, n = 20; ALF, n = 45; ACLF, n = 20; CLD; n = 15, patients with septic shock with no underlying cirrhosis/liver disease [sepsis], n = 20).

In ALF patients, there is an increase in the proportion of circulating CD4^+^ T cells (median 64.01%; IQR, 56.51%−71.61%) when compared to healthy (47.17%; 45.17%−58.12%) (*P* = .003) and pathologic controls (CLD 65.64%; IQR, 51.9%−74.07%), ACLF (57.30%; IQR, 46.54%−69.56%), and sepsis patients (70.18%; IQR, 58.61%−77.11%) (Figure 1*A*). No significant changes were seen in the proportion of circulating CD8^+^ T cells in ALF when compared to HC and pathologic control groups (Figure 1*A*). The distribution of naïve and memory subsets is markedly different, highlighted by a diminished proportion of memory population (CD4^+^CD45RO^+^CD45RA^−^) and an increased proportion of naïve cells (CD4^+^CD45RO^−^CD45RA^+^) in ALF compared with HC subjects (ratio memory to naïve: 0.4513%; IQR, 0.3328%−0.7557% vs 0.7825%; IQR, 0.5961%−1.338; *P* = .001) (Figure 1*B*). The reduction in the memory population was predominantly in the effector memory T-cell subset (CD4^+^CCR7^−^CD45RO^+^) (*P* = .002) (Figure 1*B*).

### Circulating CD4^+^ T Cells in Acute Liver Failure Are Characterized by an Immunosuppressive Cytotoxic T-Lymphocyte−Associated Protein 4−Positive Phenotype

**Figure 2:**
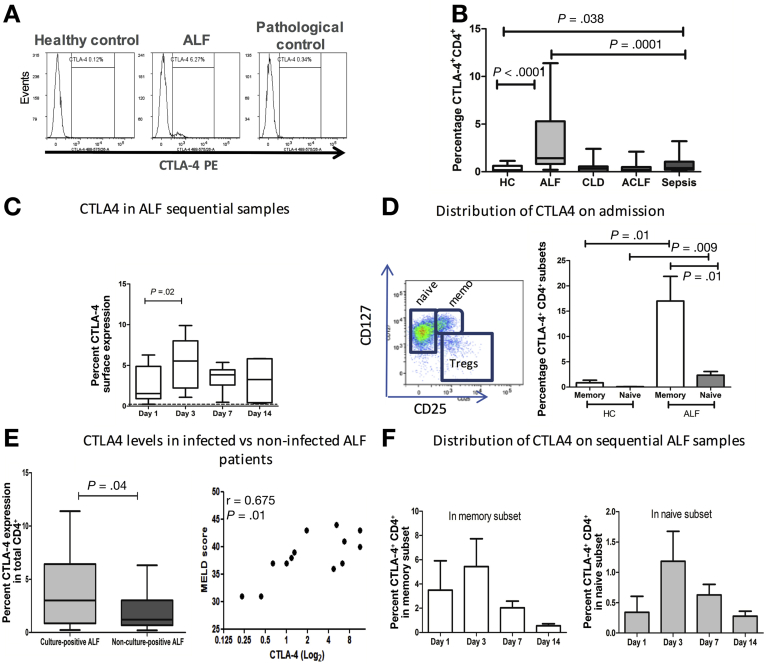
Percentages of CTLA4-expressing CD4^+^ T cells are elevated in ALF patients. (*A*) Representative flow cytometry plots to determine CTLA4-expressing CD4^+^ T cells in HC (*left*), ALF (*middle*), and pathologic controls (*right*). (*B*) Data show that percentages of circulating CD4^+^ T cells expressing CTLA4 are significantly elevated in ALF compared to HCs *(P* < .0001). (*C*) CTLA4 levels were determined in sequential samples at days 3 (n = 7), 7 (n = 8), and 14 (n = 5) after admission and compared to HC levels, represented by the dashed line. (*D*) *Left*: Representative plot to define CD3^+^CD4^+^ regulatory T cells, naïve, and memory subsets using CD25 and CD127 markers. *Right:* Distribution of CTLA4 expression in different CD4^+^ T cell subsets, mainly naïve and memory subsets on day 1 of submission (n = 15). (*E*) CTLA4 expression was assessed in ALF patients who developed infections (n = 11) and the ones who did not develop infections (n = 23). (*F*) Distribution of CTLA4^+^CD4^+^ among memory and naïve T-cell subsets assessed in longitudinal samples on days 1 (n = 6), 3 (n = 7), 7 (n = 8), and 14 (n = 5) after admission.

**Supplementary Figure 2:**
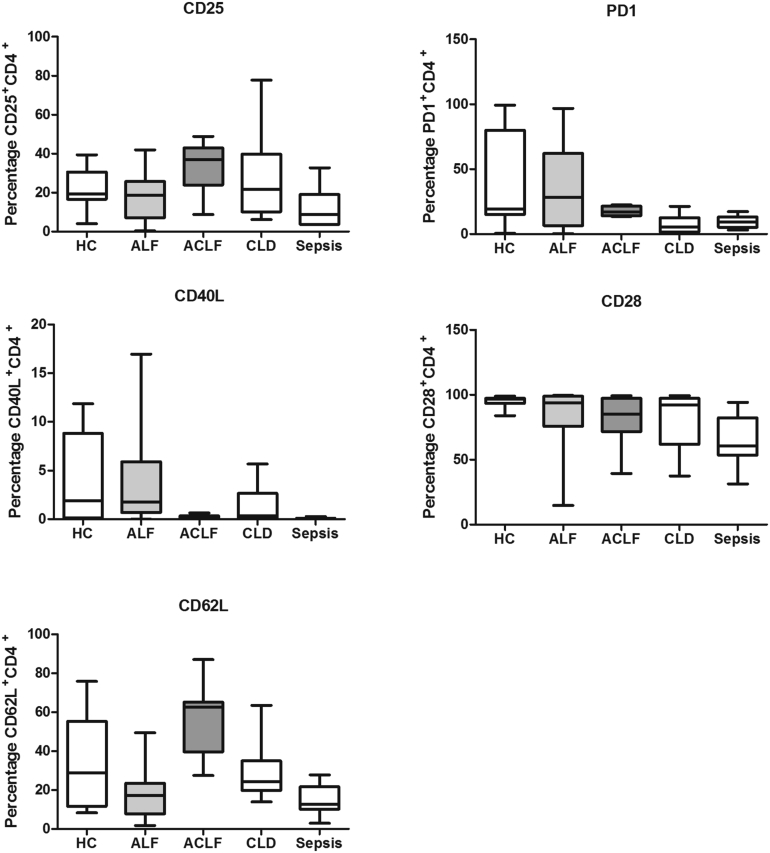
Peripheral blood mononuclear cells (PBMCs) were analyzed for surface expression of co-stimulatory (CD28, C62L) and inhibitory molecules (PD1, CD25 and CD40L) in CD3^+^CD8^−^CD4^+^ T cells by flow cytometry (HC, n = 20; ALF, n = 45; CLD; n = 15, ACLF, n = 20; nonhepatic septic shock [sepsis], n = 20).

We assessed the surface expression of activation and inhibitory markers of T-cell function. Compared to ACLF, CLD patients, and healthy controls, CTLA4 surface expression was markedly elevated in CD4^+^ T cells from ALF patients on admission to the liver intensive care unit (*P* < .0001) (Figure 2*A* and *B*). The increase in CTLA4 expression is detected in both AALF and non-AALF patients (1.390%; IQR, 0.6800%−6.418% in AALF and 1.985%; IQR, 1.020%−4.470% in non-AALF compared to 0.1750%; IQR, 0.0325%−0.6225% in healthy controls). In line with previous reports, septic shock patients also showed high levels of CTLA4 expression when compared to healthy individuals (Figure 2*B*). However, levels in septic shock patients were significantly lower that detected in ALF patients (*P* = .001). Analyses of CTLA4 expression in sequentially collected ALF samples showed that the proportion of CTLA4 expressing CD4^+^ T cells remained significantly higher than healthy controls throughout the course of admission (Figure 2*C*). At day 3 post-admission, CTLA4 levels of expression peaked, 2.5-fold higher than day-1 levels (*P* = .02) (Figure 2*C*). Detailed immunophenotypic analyses of CD4^+^ T cells revealed no significant differences in the frequencies of cells expressing CD25, PD1, CD40L CD28, and CD62L among CD4^+^ T cells in the studied groups (Supplementary Figure 2).

### Distribution of Cytotoxic T-Lymphocyte−Associated Protein 4 Expression in Different CD4^+^ T-Cell Subsets

We extended the observations reported here and investigated the distribution of CTLA4 expression on the CD4^+^ naïve and memory T-cell subsets distinguished based on expression of CD25, CD127, and CD45RA markers. The distribution of CTLA4 expression differed among ALF CD4^+^ T-cell subsets compared to HC. As illustrated in Figure 2*D*, CTLA4 expression was elevated in all CD4^+^ T cells, most marked in the memory (CD25^−^CD127^−^CD45RA^−^) when compared to the naïve (CD25^low^CD127^+^CD45RA^+^) subset. In line with previously published studies in systemic inflammatory pathologies,^31^ levels were also high in the T-regulatory subset (CD25^+^CD127^−^) (Supplementary Figure 4).

### CD4^+^ Cytotoxic T-Lymphocyte−Associated Protein 4−Positive T Cells Correlate With Disease Severity and Infectious Complications in Acute Liver Failure

**Supplementary Figure 3:**
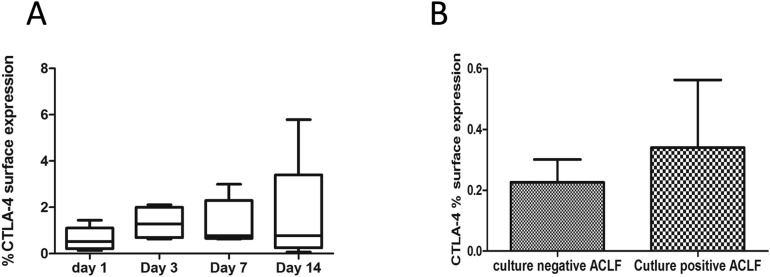
Distribution of CTLA4^+^CD4^+^ in ACLF. (*A*) Longitudinal distribution of CTLA4 expression in sequential samples on days 1 (n = 5), 3 (n = 5), 7 (n = 4), and 14 (n = 5) after admission. (*B*) CTLA4 expression in culture-negative noninfected (n = 12) and culture-positive−infected ACLF (n = 6) patients determined by flow cytometry.

**Supplementary Figure 4:**
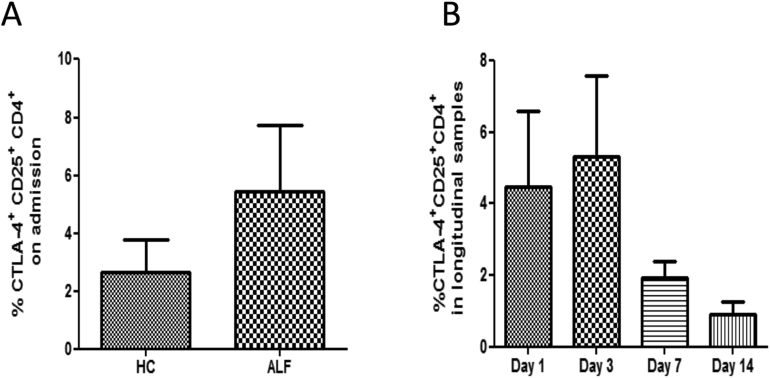
Distribution of CTLA4 surface expression in the CD127^low^CD25^high^ T regulatory cells. (*A*) On day 1 of admission (n = 15) and (*B*) in longitudinal samples on days 1 (n = 6), 3 (n = 7), 7 (n = 8), and 14 (n = 5) after admission.

**Supplementary Figure 5:**
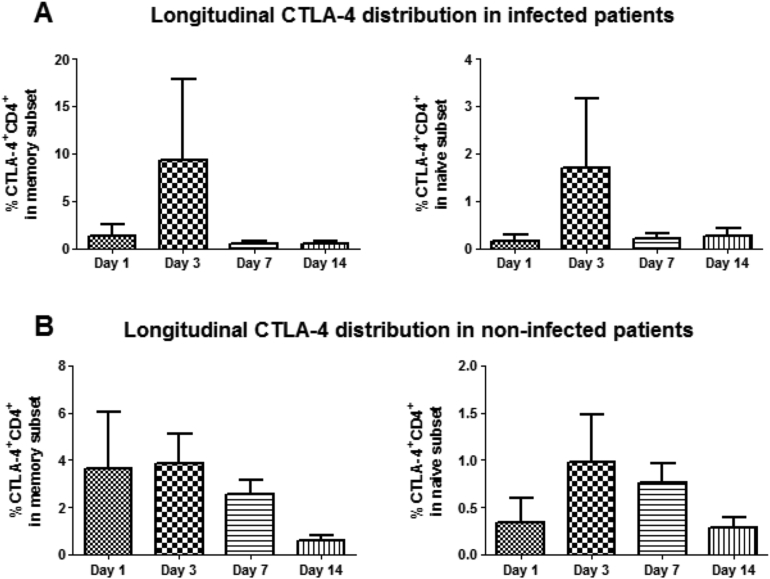
Distribution of CTLA4 surface expression in memory and naïve CD4^+^ T-cell subsets in sequential samples of ALF patients who (*A*) developed infections and (*B*) the ones who did not develop infections. *Left panels:* Distribution of CTLA4 expression in memory CD4^+^ T-cell subset. *Right panels:* Distribution of CTLA4 expression in the naïve CD4^+^ T-cell subset.

CTLA4 expression by CD4^+^ T cells was higher in patients who developed culture positive infections compared to noninfected patients (*P* = .04) (Figure 2*E*). When assessed for correlation with their corresponding clinical parameters, patients sampled on day 1 of admission who later developed culture-positive infection had elevated frequency of CTLA4^+^CD4^+^ T cell, which correlated positively with Model for End-Stage Liver Disease score (*r* = 0.675, *P* = .01) (Figure 2*E*) and ammonia (*r* = 0.771, *P* = .07) (Table 1). Analyses of the distribution of CTLA4 expression among naïve and memory subsets in patients after admission revealed that percentage of memory cells expressing CTLA4 peaked on day 3 after admission (Figure 2*E*), particularly within the infected cohort (1.330%; IQR, 0.190%−2.470% on day 1 compared to 9.320%; IQR, 0.800%−17.84% on day 3) (Supplementary Figure 5). No differences were noted in the distribution of CTLA4 when infected ACLF groups were compared to noninfected ACLF (Supplementary Figure 3*A* and *B*).

**Table 1:** Clinical and Physiological Characteristics of Culture-Positive Infected and Noninfected Acetaminophen Acute Liver Failure Patients

Parameter	Infected AALF	Noninfected AALF
Patients, n	11	24
Age, *y*	31.00 (27.00−49.00)	36.00 (27.00−48.00)
WBC, *×10*^*9*^*/L*	6.700 (5.910−8.130)	8.710 (5.925−11.61)
Monocytes, *×10*^*9*^*/L*	0.2000 (0.1300−0.2600)	0.3200 (0.1250−0.6050)
Lymphocytes, *×10*^*9*^*/L*	0.5100 (0.3000−0.7400)	0.7400 (0.3200−1.105)
SIRS score	3.000 (2.000−4.000)	3.000 (2.000−4.000)
MELD score	39.00 (37.00−43.00)	39.00 (31.00−41.00)
Bilirubin, *μmol/L*	70.00 (44.00−140.0)	90.00 (69.00−162.0)
INR	4.040 (2.870−6.250)	4.480 (2.480−6.340)
Creatinine, *μmol/L*	259.0 (90.00−360.0)	93.00 (63.00−239.5)
Urea, *mmol/L*	11.30 (5.700−14.00)	7.000 (4.900−8.700)
AST, *IU/mL*	4703^a^ (2521−8861)	1665 (951.5−3756)
Ammonia, *μmol/L*	87.50 (54.75−138.5)	99.00 (67.50−150.3)
Encephalopathy score	3.000^b^ (3.000−4.000)	2.000 (1.000−3.000)
CTLA4, *%*	3.005^b^ (0.8575−6.418)	1.200 (0.6750−3.015)
Outcomes,^c^ n		
OLT	2	8
Survivors	6	16
Nonsurvivors	3	0

NOTE. Values are median (IQR) unless otherwise noted.

ALD, acute liver disease; AST, aspartate aminotransferase; INR, international normalized ratio; MELD, Model for End-Stage Liver Disease; OLT, orthotopic liver transplantation; NA, not applicable; ND, not determined; SIRS, systemic inflammatory response syndrome criteria score.

a *P* = .01.

b *P* < .002, compared to noninfected.

c Outcomes at 28 days post admission.

### Defects in CD4^+^-Mediated T-Cell Responses Are Restored Through Blocking Cytotoxic T-Lymphocyte−Associated Protein 4

**Figure 3:**
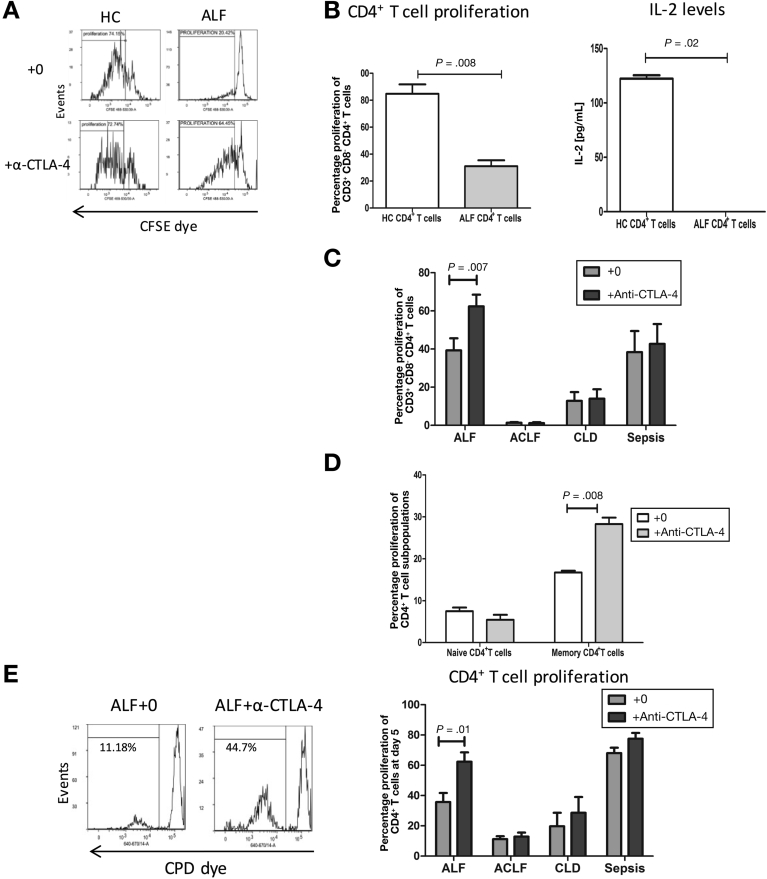
Attenuated ALF recall responses to HLA class II−restricted T cell epitopes. (*A*) peripheral blood mononuclear cells from HCs (n = 5) and ALF (n = 5) were labeled with carboxyfluorescein succinimidyl ester and stimulated for 6 days with HLA class II-restricted T-cell peptide pool at 10 μg/mL and examined in vitro in the presence or absence of α-CTLA4 blocking antibody (10 μg/mL). Recall responses induced in the CD3^+^CD8^−^CD4^+^ T-cell population were assessed and expressed as percentages of proliferating cells. Representative flow cytometry histograms of the proliferation responses (shown within the *marker gate*) in HCs (*left*) and ALF (*right*) and assessed in the absence (*top panels*) or the presence (*bottom panels*) of α-CTLA4 antibody. (*B*) The resulting proliferation (*B, left*) and IL2 production (*B, right*) were determined by flow cytometry and enzyme-linked immunosorbent assays, respectively. (*C*) Proliferation in CD4^+^ T cells in ALF (n = 8), CLD (n = 4), ACLF (n = 4), and sepsis (n = 6) after neutralization of CTLA4. (*D*) CTLA4 blockade using α-CTLA4 antibody restored proliferation particularly in the memory subset (n = 5). (*E*) Representative flow cytometry histograms of proliferation of isolated CD4^+^T cells from ALF patients labeled with CPD eFluor 670 and co-cultured with monocyte-derived healthy donor DCs (1:40 DC/CD4^+^ T-cell ratio) in the presence of α-CD3 antibody (*left histogram*). Data show the resulting proliferation at day 5 (*right panel*) in the presence or absence of α-CTLA4 in ALF (n = 8), CLD (n = 4), ACLF (n = 4), and sepsis (n = 6).

**Supplementary Figure 6:**
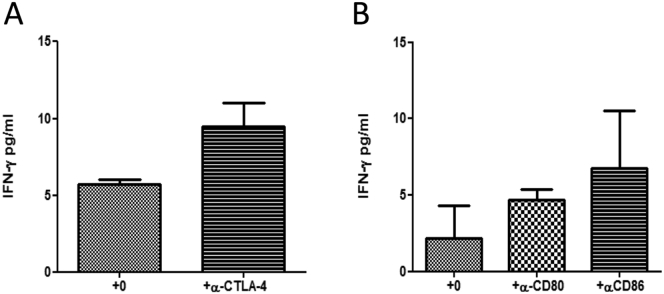
IFN-gamma levels assessed by MSD. (*A*) Supernatants from cultures in the presence or absence of α-CTLA4 blocking antibody. (*B*) IFN-gamma levels in cultures preconditioned in sera treated with α-CD80 and CD86 (*right panel*) (n = 8).

To investigate whether phenotypic changes reflect a change in the functional capacity in CD4^+^ T cells in ALF, we assessed the proliferative capacity of CD4^+^ T cells using both antigen-dependent and independent systems. Firstly, in response to major histocompatibility complex class II−restricted recall antigens, we reveal that T-cell proliferation and IL2 secretion were significantly reduced in ALF (*P* = .008 and *P* = .02, respectively) when compared to HC (Figure 3*A* and *B*). T-cell proliferation was reversed after blockade of the CTLA4 pathway (*P* = .007) (Figure 3*C*). Significant increases in T-cell proliferation after CTLA4 pathway blockade in ACLF or CLD patient groups was not detected (Figure 3*C*). IL2 production was induced after CTLA4 blockade in ALF (12.50 pg/mL; IQR, 9.810−40.25 pg/mL) (*P* = .01). IFN gamma levels were also restored, but did not reach statistical significance (Supplementary Figure 6*A*). Furthermore, the improvement in proliferative capabilities was most pronounced in memory CD4^+^CD45RO^+^ subset (Figure 3*D*). Then, we determined whether CD4^+^ T cells from ALF patients responded to T-cell receptor−mediated activation with α-CD3 antibody in the presence of allogeneic healthy donor-monocyte−derived DCs. CD4^+^ T cells isolated from HCs underwent more cell divisions in co-culture with allogeneic DCs after α-CD3 antibody stimulation than the high expressing CTLA4 CD4^+^ T cells derived from ALF patients (Figure 3*E*). To confirm the suppressive effect of CTLA4 in CD4^+^ T cells from ALF patients, co-cultures were carried out in the presence of anti-CTLA4 antibody. Blocking CTLA4 activity restored CD4^+^ T-cell proliferation (*P* = .03) and IL2 production (114.8 pg/mL; IQR, 61.51−172.4 pg/mL compared to 244.1 pg/mL; IQR, 165.9−294.6 pg/mL after blocking CTLA4) (*P* = .04). Analyses of CD4^+^ T-cell function after CTLA4 blockade revealed no significant differences in the ACLF and CLD patient groups (Figure 3*E*).

### Soluble Circulating Mediators in Acute Liver Failure Regulate Cytotoxic T-Lymphocyte−Associated Protein 4 Expression

**Figure 4:**
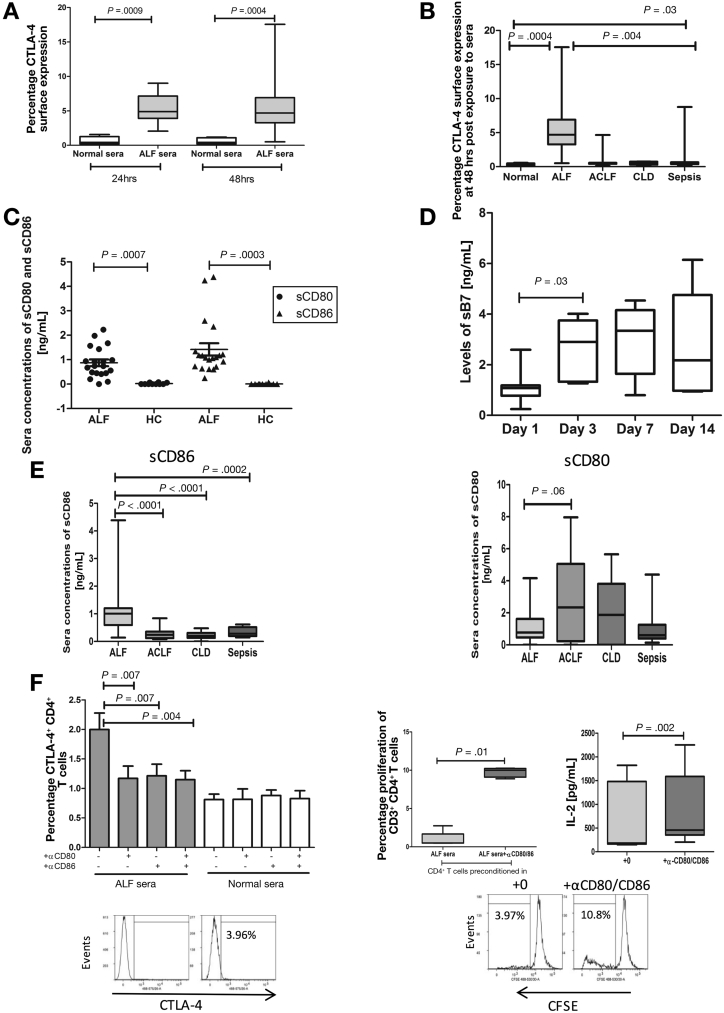
Effect of circulating soluble mediators on CTLA4 expression. (*A*) CTLA4 levels in purified CD4^+^ T conditioned in media supplemented with HC (n = 12) or ALF sera (n = 14) were analyzed using flow cytometry after 24-hour and 48-hour culture. (*B*) CTL4 levels in purified CD4^+^ T cells conditioned in media supplemented with sera from ACLF (n = 15), CLD (n = 6) or sepsis (n = 10) sera in comparison to levels induced by culture in the presence of ALF and normal sera. (*C*) Detection of levels of soluble costimulatory molecules sCD80 and CD86 in sera samples from ALF (n = 20) and HC (n = 10) by enzyme-linked immunosorbent assay (ELISA). (D) sCD80 and sCD86 in sequential samples on days 1 (n = 12), 3 (n = 6), 7 (n = 7), and 14 (n = 5) after admission. (*E*) Circulating levels of sCD86 and sCD80 in sera from ALF (n = 25), ACLF (n = 20), CLD (n = 15), and sepsis (n = 20) patients determined by ELISA. (*F*) Healthy and ALF sera were preincubated with anti-CD80 or CD86 to block soluble CD80 or CD86, respectively. Results are representative of 8 independent experiments. Representative histograms gated on CD3^+^ T cells demonstrating proliferation percentages of carboxyfluorescein succinimidyl esterlabeled CD4^+^ T cells preconditioned in ALF sera in the absence (*left histogram*) or presence (*right histogram*) of pretreatment with anti-CD80 or CD86 blocking antibodies. Proliferation (*middle panel*) and IL2 secretion (*right panel*) are from 5 independent experiments.

In view of the importance of the inflammatory microenvironment in modulating immune cell function in ALF,^4^, ^6^, ^7^, ^32^ we investigated the effects of sera, derived from ALF patients, on CTLA4 expression in CD4^+^ T cells from HC. Exposure to sera from ALF patients resulted in up-regulation of CTLA4 expression on CD4^+^ T cells after 24 and 48 hours in culture (*P* = .01) (Figure 4*A*). Similar to the effect of sera from ALF patients, we report an elevation in CTLA4 expression after exposure to sera from sepsis patients. However, these levels were still significantly lower than what we detect after exposure to sera derived from ALF patients (Figure 4*B*). No effect on CTLA4 up-regulation was detected in cultures of CD4^+^ T cells in sera derived from ACLF and CLD patients (Figure 4*B*).

In order to provide a mechanistic explanation for the activation of the CTLA4 pathway in ALF, we sought to investigate the role of circulating sB7 molecules (sCD80 and sCD86). Here, we detected a marked elevation in circulating concentrations of sCD80 and sCD86 in ALF compared with sera from ACLF, CLD, and HCs (Figure 4*C, E*). Similar to the CTLA4 levels, sB7 concentrations peaked at day 3 of admission (*P* = .03) and remained persistently elevated until day 14 (Figure 4*D*).

We hypothesized that high levels of circulating sB7 detected in ALF are responsible for the activation of the CTLA4 pathway in circulating CD4^+^ T cells. To test this hypothesis, we assessed the effect of neutralizing sCD80 and CD86 in ALF sera before exposure to CD4^+^ T cells from HCs. As shown in Figure 4*F*, elevations in CTLA4-expressing CD4^+^ T cells were significantly reduced, while proliferation and IL2 secretion were augmented (*P* = .01 and *P* = .002, respectively) after neutralization of sCD80 and sCD86 in sera derived from ALF patients when compared to HC (Figure 4*F*). Similarly, we report an increase in levels of IFN gamma, but this did not reach statistical significance (Supplementary Figure 6*B*).

### Soluble B7s Are Secreted by Injured Hepatocytes in Acute Liver Failure

**Figure 5:**
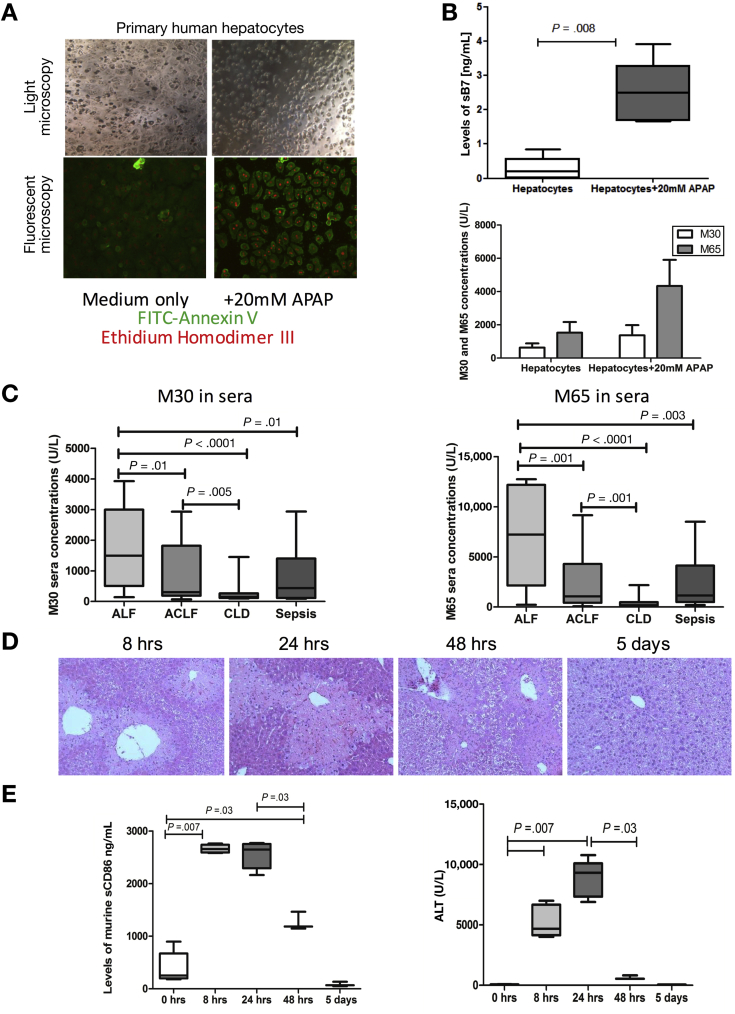
Human and murine sources of soluble sCD80 and sCD86. (*A*) Hepatocytes were first assessed for apoptosis and necrosis. Apoptotic cells are stained *bright green* and necrotic *bright red*. (*B*) Primary human hepatocytes were tested for their ability to secrete sCD86 hepatocytes after APAP treatment. Supernatants from 24 hours post APAP-treated hepatocytes were assessed by enzyme-linked immunosorbent assays (ELISA) for concentrations of sCD86 (*top panel*) and for soluble cell death markers (M30 and M65) (*bottom panel*). (*C*) Circulating levels of M30 (*left panel*) and M65 (*right panel*) in ALF (n = 25), ACLF (n = 20), CLD (n = 15), and sepsis (n = 20) patients determined in sera by ELISA. (*D*) Representative H&E-stained histology sections of liver tissue from murine model of APAP-induced liver injury highlighting different stages in the evolution and recovery from APAP-induced liver injury. Liver injury initiation (characterized by centrilobular hepatocyte necrosis) (8 hours), peak (24 hours), and resolution (within 5 days). (*E) Left:* Levels of sCD86 measured in APAP-injury murine sera at 0 hours, 8 hours, 24 hours, 48 hours, and 5 days post APAP-induced liver injury and (*right*) corresponding alanine aminotransferase (ALT) sera levels (n = 5 per group).

In order to investigate the source of the increased circulating levels of sB7 molecules in the circulation, we assessed the ability of hepatic parenchymal (hepatocytes and biliary epithelial cells) and nonparenchymal cells (Kupffer cells, monocytes) to release sB7 after in vitro and in an in vivo model of APAP-induced acute liver injury. Although immune cells, HSECs, and biliary epithelial cells in ALF did not release increased concentrations of sB7, APAP-treated necrotic primary human hepatocytes (Figure 5*A*) released high levels of sB7 (Figure 5*B*). No detectable levels of sB7 ligands were seen in all tested activated and nonactivated immune and nonimmune cell types. Epithelial cell death was further validated by assessing levels of cell death markers (caspase-cleaved [CK-18 {M30}] and total cytokeratin [CK-18 {M65}]) (Figure 5*B*). In addition to elevated levels of sB7, primary human hepatocytes secreted elevated levels of M30 and M65 after APAP-treatment (Figure 5*B*). We further assessed sera concentrations of M30 and M65 in ALF patients. Levels of M30 and M65 were significantly elevated in ALF patients (Figure 5*C*) and strongly correlated with release of sCD86 (M30: *r* = 0.492, *P* = .01; M65: *r* = 0.420, *P* = .03).

Having demonstrated in vitro that APAP-treated hepatocytes represented the main source of sB7, we postulated that sB7 are released as early mediators from damaged hepatocytes and they play a key role in initiating and maintaining elevated CTLA4 expression levels on circulating CD4^+^ T cells. We examined this in vivo using a time-course APAP-induced acute liver injury murine model^33^ (Figure 5*D*). Consistent with the in vitro findings, APAP injury resulted in the release of increased concentrations of sB7, which were detected in the sera as early as 8 hours post APAP treatment (initiation phase) (*P* = .007), remained elevated between 8 and 24 hours (peak liver injury), and significantly decreased at 48 hours onward (resolution phase) (*P* = .03) to return to baseline levels after 5 days (Figure 5*E*).

### Plasma Exchange Modulates Cytotoxic T-Lymphocyte−Associated Protein 4 Activation Through Clearance of Circulating Soluble B7 Molecules in Acute Liver Failure

**Figure 6:**
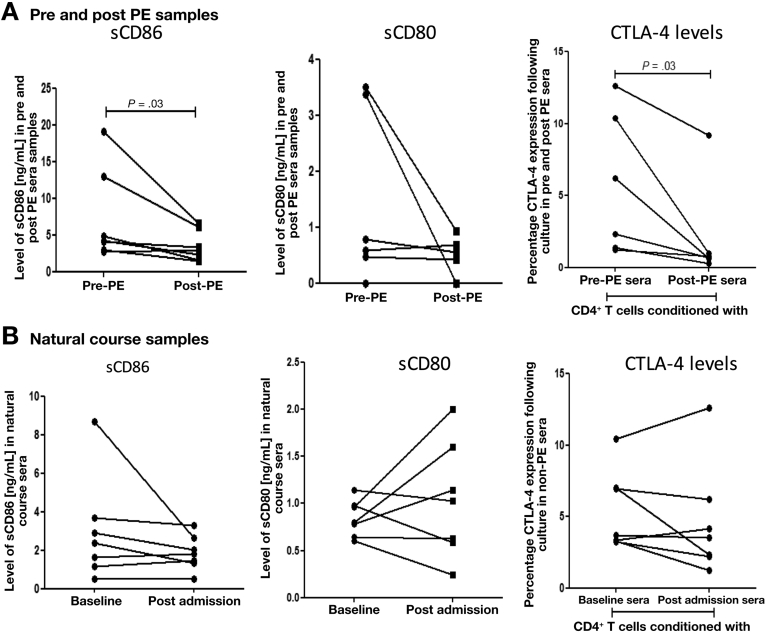
PE results in the removal of circulating soluble CD80 and sCD86 and the reduction of CTLA4 levels on CD4^+^ T cells. (*A*) Levels of soluble CD86 (*left panel*) and sCD80 (*middle panel*) were assessed by enzyme-linked immunosorbent assays (ELISA) in sera from pre- and post-PE patients and (*B*) in natural course samples. (*A) Right*: CTLA4 levels were assessed by flow cytometry after cultures of purified CD4^+^ T cells from HCs in media supplemented with sera from pre- and post-PE patients (n = 7) and (*B) Right:* sera from natural course patients group who did not undergo PE (n = 7).

PE has been previously reported to be an important therapeutic intervention capable of modulating innate immune responses in ALF, but its effects on adaptive immunity are not known.^12^ We assessed the levels of sCD80 and sCD86 and showed that these were significantly reduced after PE (Figure 6*A*). In contrast, no reductions in sB7 levels were detected in ALF patients who did not undergo PE (Figure 6*B*). To assess whether these reductions in circulating titers of sB7 molecules attenuate CTLA4 expression, we performed in vitro cultures quantifying the CTLA4 expression on CD4^+^ T cells after incubation of HC-derived CD4^+^ T cells in sera obtained from ALF patients before (pre-PE) and after (post-PE) PE. Exposure of CD4^+^ T cell to pre-PE sera resulted in a significant increase in CTLA4 levels, whereas exposure to post-PE sera did not (Figure 6*A*). These results indicate that PE sera drive a markedly different phenotype of the CD4^+^ T-cell population characterized by reduced levels of CTLA4.

### Discussion

This study identifies adaptive immune dysfunction, mediated through CTLA4 that is triggered by soluble co-stimulatory sB7 molecules released from the acutely inflamed liver in patients with ALF. Here, we identify not only a numerical reduction in circulating lymphocytes, but also an increased proportion of CD4^+^ T cells bearing the inhibitory, CTLA4-positive phenotype specifically in AALF and non-AALF patients and not in other acute and chronic hepatic inflammatory diseases. CTLA4 expression was particularly elevated in memory CD4^+^ T cell, an immune cell subset responsible for protective immunity against microbial pathogens.^34^ These findings are likely to be of pathogenic significance in ALF, given that higher level of CTLA4-expressing cells on admission are detected in patients who proceed to develop culture-positive secondary infections. Therefore, the elevated CTLA4 levels in the memory CD4^+^ T-cell compartment is likely to reflect an impaired capacity to protect against re-infection and a defect in producing robust effector function responses upon microbial challenge. The increased proportion of CTLA4-bearing cells in ALF indicates a central role for CTLA4 pathway in the predisposition to infection and that blockade of this immunosuppressive pathway may be beneficial in restoring antimicrobial responses. We reveal that blocking activation of the CTLA4 pathway significantly restores antigen-specific responses, particularly in the memory CD4^+^ T-cell subset; with similar findings reported in CTLA4-expressing memory CD8^+^ T cells.^35^ It is interesting to note that infected ALF patients had persistent elevated CTLA4 expression on CD4^+^ T cells. In this regard, several experimental and clinical studies showed the involvement of CTLA4-mediated negative regulation in infections.^36^, ^37^ CTLA4 was reported to be expressed at higher levels in patients with sepsis than in critically ill nonsepsis patients and was associated with impairment in T-cell responses.^38^, ^39^ Furthermore, in severe sepsis, T-cell apoptosis and dysfunction were associated with an up-regulation of CTLA4 on CD4^+^ T cells.^40^, ^41^ Similarly, increased levels of CTLA4 were implicated in viral infection-associated complications.^42^, ^43^ Accordingly, in murine models of bacterial and fungal sepsis, blocking CTLA4 improved pathogen clearance and survival.^16^, ^17^

The immune regulatory functions of the B7/CD28/CTLA4 pathway are well recognized in acquired immunodeficiency and autoimmune disorders.^44^, ^45^ It is known that membrane-bound CD80 and CD86 represent the shared ligands for CD28 and CTLA4.^46^ However, it has also been shown that soluble forms of B7 molecules represent an alternative powerful mechanism by which antigen-presenting cells could modulate the signals normally generated via membrane-bound forms of B7. Experimental studies indicate that recombinant sB7 molecules represent a putative mechanism for modulating T-cell responses through either inhibition or enhancement of immune responses.^47^, ^48^ In this study, we provide a novel mechanistic explanation as to the pathogenesis of adaptive immune dysfunction in patients with ALF. Here, we report a marked and persistent elevation in circulating titers of sB7 molecules, in particular sCD86, which strongly correlate with indices of severity of acute hepatic injury and more specific markers of hepatocellular death (M30/M65). In view of these findings, we hypothesized that the origin and release of these molecules was from the acutely injured liver. We screened for sB7 release in parenchymal and nonparenchymal cells and we identify that the likely origin of sB7 molecules is from hepatocytes that have undergone necrotic cell death after APAP administration. This finding is corroborated by in vivo data from the murine model of APAP-induced liver injury, where highest circulating titers of sB7 are detected at peak hepatoxicity. Taken together, we highlight a novel role for sB7 molecules, released after hepatocyte cell death, that negatively regulate the adaptive immune responses through activation of the CTLA4 pathway. Although our data support that epithelial cell death is responsible for increased levels of sB7, in particular sCD86, it cannot be excluded that during ALF, renal replacement therapy alone would be a sufficient modality in clearing the sudden increase in concentrations of these circulating proteins from the circulation. This is supported by our findings in which we report that PE does result in significant reductions in concentrations of sCD86 levels in patients with acute liver injury. Additional studies are required to determine whether this mechanism of adaptive immune dysfunction is common to other hepatic and nonhepatic systemic inflammatory pathologies characterized by sudden and overwhelming acute tissue injury.^9^

This work has identified a novel therapeutic target to reverse immune dysfunction in patients with ALF. CTLA4 inhibitory strategies are established immune checkpoint inhibitors in malignant and nonmalignant inflammatory pathologies.^16^, ^25^, ^49^ However, there would be concern about the use of this immunotherapeutic strategy in ALF (eg, ipiluminab), given its significant side-effect profile (eg, colitis, dermatitis, and autoimmune hepatitis^24^) and potential risk in impairing hepatic regenerative responses.

PE has recently been shown to be of benefit in patients with ALF through clearance of damage-associated molecular patterns and other intracellular products released from the injured liver.^12^ Given the fact that sB7 molecules, released from necrotic hepatocytes, are responsible for activation of the CTLA4 pathway, we postulated that PE would attenuate CTLA4 levels through removal of sB7 molecules. Here we demonstrate that PE significantly reduces circulating titers of sB7 molecules, in particular sCD86, and CTLA4 expression. These data indicate that PE has beneficial effects in modulating not only innate, but also adaptive immune responses and might therefore represent a plausible therapeutic strategy to restore adaptive immune responses and reverse immune paresis, while the injured liver undergoes regeneration in ALF. Future prospective studies are required to address this important issue.

In summary, we show that activation of the CTLA4 pathway is responsible for adaptive immune dysfunction, immune paresis, and infection susceptibility in patients with ALF. Furthermore, we highlight the role of sB7 molecules, released from acutely injured liver, in up-regulating this suppressive pathway in circulating CD4^+^ T cells. PE might represent a credible immunotherapeutic strategy aimed at restoring adaptive immune responses against microbial pathogens by reducing concentrations sB7 molecules and CTLA4-bearing T cells.

## Figures and Tables

**Supplementary Figure 1:**
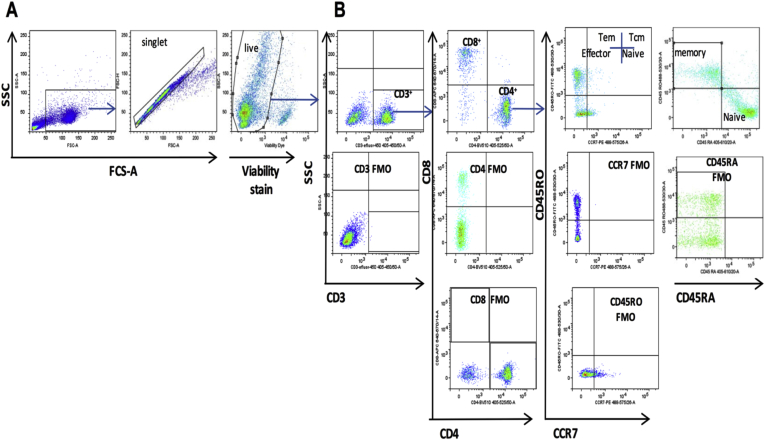
Gating strategy to define lymphocyte subsets. (*A*) Representative dot plots of the gating strategy. Lymphocytes were first gated according to the forward and side scatter profile. Doublets were excluded from the analyses using side scatter area vs width discrimination. Dead cells, which were determined by positive staining for the cell viability dye, were then excluded. (*B*) CD3 then CD4 and CD8 markers were used to determine the lymphocyte primary populations, which were then gated into subsets according to the corresponding fluorescence-minus-one (FMO) control (*middle and bottom panels*). FCS-A, forward scatter area; SSC, side scatter.

**Supplementary Table 1:** Demographic and Clinical Parameters of Acute Liver Failure, Chronic Liver Disease, Acute-on-Chronic Liver Failure, and Nonhepatic Septic (Sepsis) Patients and Healthy Controls

Parameter	HC	ALF	CLD	ACLF	Septic
Patients, n	20	45	15	20	20
Age, *y*	33.00 (27.25−36.75)	35.00^a^^,^^b^^,^^c^^,^^d^ (26.50−48.5)	59.00 (44.5−61.60)	51.70 (39.00−61.50)	48.00 (32.25−61.25)
Etiology, n	NA	Acetaminophen, 35 Autoimmune, 2 Seronegative, 3 Drug-induced liver injury, 5	Alcoholic liver disease, 13 HCV, 1 Wilson’s disease, 1	Alcohol-related disease, 16^e^ NAFLD, 3^e^ Cryptogenic cirrhosis, 1^e^	Pneumococcal, 7 Urologic, 2 Streptococcal cellulitis, 3 Streptococcal meningitis, 1 Liver abscess, 1 Fournier’s, 1 Chest sepsis, 5
WBC, *×10*^*9*^*/L*	NA	6.040^a^^,^^b^ (9.880−8.130)	6.110 (4.000−5.200)	8.100 (4.300−13.55)	9.335 (5.528−13.52)
Monocytes, *×10*^*9*^*/L*	NA	0.2800^a^^,^^b^^,^^c^ (0.1300−0.5100)	0.6000 (0.3000−0.4200)	0.5750 (0.4500−1.050)	0.3350 (0.1300−0.6850)
Lymphocytes, *×10*^*9*^*/L*	NA	0.6800^a^^,^^b^^,^^c^ (0.3300−1.190)	1.400 (0.700−1.770)	0.6700 (0.4525−1.350)	0.6400 (0.275−0.9300)
SIRS score	NA	3.000^∗,^^c^ (2.000−4.000)	ND	1.500 (1.000−2.000)	4.000 (3.00−5.000)
MELD score	NA	38.00^∗,^^a^^,^^b^ (31.00−40.00)	11.00 (9.000−16.00)	22.50 (17.80−26.25)	NA
Child-Pugh	NA	NA	8.000 (6.000−9.000)	10.00 (9.000−13.00)	NA
SOFA score (CLIF−SOFA score in ACLF)	NA	13.50^a^^,^^d^ (7.750−17.00)	NA	10.00 (8.000−17.00)	6.000 (4.000−9.000)
Bilirubin, *μmol/L*	NA	94.00^a^^,^^b^^,^^c^^,^^d^ (57.00−161.0)	28.00 (20.00−69.00)	218.0 (110.0−425.8)	14.50 (11.25−25.00)
INR	NA	3.840^a^^,^^b^^,^^c^^,^^d^ (2.450−6.295)	1.300 (1.200−1.440)	1.655 (1.475−1.950)	1.430 (1.190−1.918)
Creatinine, μmol/L	NA	154.0^a^^,^^b^^,^^c^ (67.00−288.0)	64.00 (61.00−75.00)	79.00 (59.00−116.0)	101.0 (75.00−116.0)
CVVH, *%* (n)	NA	60 (27)	NA	45 (9)	40 (8)
Urea, *mmol/L*	NA	7.200^a^^,^^b^ (5.300−13.10)	4.300 (3.100−5.350)	5.500 (4.050−7.325)	5.850 (3.600−8.100)
AST, *IU/mL*	NA	3584^a^^,^^b^^,^^c^^,^^d^ (1010−6073)	47.00 (39.00−245.0)	92.00 (58.00−128.0)	49.50 (34.00−241.0)
Ammonia, *μmol/L*	NA	94.00 (65.00−127.0)	89.00 (88.00−90.00)	ND	ND
Outcomes,^f^*%* (n)	NA	Spontaneous survivors 69 (31) OLT 24.4 (11) Nonsurvivors 6.6 (3)	Survivors 100 (15)	Spontaneous survivors 60 (12) OLT 15 (3) Nonsurvivors 25 (5)	Survivors 95 (19) Nonsurvivors 5 (1)

NOTE. Values are medians (IQR), unless otherwise noted.

AST, aspartate aminotransferase; CLIF−SOFA, chronic liver failure−sequential organ failure assessment; CVVH, continuous veno-venous hemofiltration; HCV, hepatitis C virus; INR, international normalized ratio; MELD, Model for End-Stage Liver Disease; NA, not applicable; NAFLD, non-alcoholic fatty liver disease; ND, not determined; OLT, orthotopic liver transplantation; SIRS score, systemic inflammatory response syndrome criteria score.

a *P* < .05.

b Significant differences comparing ALF to CLD patients.

c Significant differences comparing ALF to ACLF.

d Significant differences comparing ALF to sepsis patients.

e Cause for decompensation in ACLF: alcoholic hepatitis (n = 11), variceal bleed (n = 2), sepsis (n = 7).

f Outcomes at 28 days post admission.

**Supplementary Table 2:** Demographic and Clinical Parameters of Pre-PE and Post-PE Acute Liver Patients

Parameter	Pre-PE	Post-PE
Patients, n	7	
Age, *y*	46 (36.5−52)	
Etiology, n		
APAP	5	
Acute Wilson’s disease	1	
Liver trauma	1	
WBC, *×10*^*9*^*/L*	11.4 (8.3−12.8)	7.2 (5.9−9.4)
Monocytes, *×10*^*9*^*/L*	0.1800 (0.1100−0.3300)	0.2400 (0.1500−0.3000)
Lymphocytes, *×10*^*9*^*/L*	0.8 (0.3−0.8)	0.4 (0.3−0.6)
SIRS score	2 (2−3)	2 (2−3)
MELD score	40 (39−40)	37 (34−38)
Bilirubin, *μmol/L*	87 (73.5−97.5)	95 (88−112)
INR	3.14 (3.01−9.12)	2.03 (1.64−2.32)
Creatinine, *μmol/L*	148 (120−301)	125 (94.5-171)
Urea, *mmol/L*	7.600 (4.800−9.400)	4.300 (2.500−6.400)
AST, *IU/mL*	5420 (3424−8834)	2196 (1214−3587)
Ammonia, *μmol/L*	58.00 (39.50−71.50)	45.00 (14.00−201.0)
Outcome		
Nonsurvivors	2	
OLT	2	
Survivors	3	

NOTE. Values represent medians (IQR), unless otherwise noted.

AST, aspartate aminotransferase; INR, international normalised ratio; OLT, orthotopic liver transplantation; MELD, Model for End-Stage Liver Disease; SIRS score, systemic inflammatory response syndrome criteria score; WBC, white blood cells.

## Supplementary Materials and Methods

### Collection of Peripheral Blood Mononuclear Cells, Isolation of CD4^+^ T Cells, and Generation of Monocyte-Derived Dendritic Cells

Peripheral blood mononuclear cells were isolated from whole blood through Ficoll-paque Plus (GE Healthcare Bio-Sciences AB, Uppsala, Sweden) gradient centrifugation according to the manufacturer’s instructions. CD4^+^T cells and CD14^+^ monocytes were isolated by negative and positive magnetic bead selection, respectively (Miltenyi Biotec, Surrey, UK) according to the manufacturer’s instructions. Purified cells were routinely tested for purity using flow cytometry and were >98%. Monocyte-derived DCs were generated from isolated CD14^+^ cells as described previously.^26^ Briefly, DCs were generated after 7 days of culture in the presence of recombinant human IL4 and granulocyte-macrophage colony-stimulating factor (eBioscience). DCs were characterized by the expression of CD1a, CD11c, HLA-DR, CD80, CD83, CD86, and CD14. On day 6, DCs were matured by addition of lipopolysaccharide from *Escherichia coli* (1 μg/mL) (Source BioScience, Nottingham, UK). DCs were cultured in RPMI medium supplemented with 10% fetal calf serum and 2 mmol l-glutamine (Invitrogen, Paisley, UK). Viable cell counts were determined by trypan blue exclusion.

### Phenotyping Using Flow Cytometry

Peripheral blood mononuclear cells were surface stained for markers using the following pre-titrated fluorochrome-labeled mouse anti-human monoclonal antibodies: CD3-eFluor 450, CD4-Brilliant Violet 510, CD45RA-Brilliant Violet 605, PD-1-Brilliant Violet 786, CD45RO-fluorescein isothiocyanate, CTLA4/C-C chemokine receptor type 7−phycoerythrin (PE), CD25 BD HorizonTM PE-CF594, CD62L peridinin chlorophyll−eFluor710 (All from eBioscience) and CD28/CD127 PE−cyanine 7, CD8/CD40L allophycocyanin (Becton Dickinson Ltd). Fluorescence minus one controls for each marker were used to set the gates to define the different subpopulations of lymphocytes. Cell Viability Dye eFluor780 was used for the exclusion of dead cells. Peripheral blood mononuclear cells were incubated in the presence of the antibodies or the corresponding fluorescence minus one controls in the dark for 20 minutes (except for C-C chemokine receptor type 7, which was incubated for 45 minutes) at 4°C. Single-color controls for compensation were performed using UltraComp eBeads (eBioscience). The single stain compensation control for the cell viability dye eFluor780 was performed using ArC beads (ThermoFisher Scientific) following the manufacture’s protocol. Twelve-color flow cytometric analyses were performed using Fortessa flow cytometer. Data were acquired using BD FACSDiva software (Becton Dickinson Ltd). A total of 10,000−20,000 gated events were analyzed for each cell population. Data were analyzed using FlowLogic software (Inivai Technologies Pty Ltd). When assessed for T-cell proliferation using CPD dye eFluor670 labeling, cells were labeled with CD3-eFluor450, CD4-Brilliant Violet 510, CD8 allophycocyanin, CD45RA-Brilliant Violet 605 and CD45RO-fluorescein isothiocyanate antibodies to assess proliferation of CD4^+^ T cells memory vs naïve populations.

### Primary Human Hepatocytes and Kupffer Cells

Cryopreserved hepatocytes were obtained from Life Technologies Ltd (Paisley, UK) and cultured following manufacturer’s instructions. Hepatocytes were first thawed using Cryopreserved Hepatocyte Recovery Medium, cell count and viability were determined using trypan blue staining. Viability was >97%. Cells were then plated using Cryopreserved Hepatocyte Plating Medium in 6 chamber slides collagen coated plates (Life Technologies Ltd, Paisley, UK) at a lot-specific predetermined seeding density (0.9 × 10^6^ cells/mL) for 4 hours to allow adherence of the cells before washing and replacing the Cryopreserved Hepatocyte Plating Medium with Williams’ E supplemented medium (Life Technologies Ltd). Hepatocytes were allowed to further adhere overnight. Cultures were then checked for their cell morphology and monolayer integrity under the microscope before being washed. Freshly supplemented media was replaced with medium only or medium containing 20 mM APAP (Sigma) for 24-hour exposure. Non-APAP controls were included. Cells were stained using apoptotic/necrotic cell kit (PromoCell GmbH) Annexin V, Ethidium homodimer III, respectively, and according to the manufacturer’s instructions. Cells were then fixed in 4% paraformaldehyde and mounted using PromoFluor antifade Reagent (PromoCell GmbH). Wide-field epifluorescence microscopy was performed on a Leica DMI6000B equipped with a Hamamatsu ORCA-ER CCD camera operated by the MetaMorph software (Molecular Devices, Sunnyvale, CA). Cryopreserved KCs were obtained from Invitrogen and were recovered in Dulbecco’s modified Eagle medium thawing and plating media (Life Technologies). KCs were seeded at a minimum density of 0.2−0.4 × 10^6^ cells/mL on 24-well CellBIND plates (VWR, Lutterworth, UK). After 3 hours of culture, the KCs were washed and media refreshed. After overnight stimulation, media was replaced with Dulbecco’s modified Eagle medium maintenance media (Life Technologies Ltd). KCs were then stimulated in the presence of lipopolysaccharide (1 μg/mL) for 2 hours, washed, then co-cultured with APAP-treated hepatocytes at a 1:4 cell ratio overnight. Supernatants from hepatocyte and KC cultures were collected for assessment of soluble B7 using enzyme-linked immunosorbent assay.

### Isolation and Culture of Human Biliary Epithelial Cells

Human biliary epithelial cells (BECs) were isolated from 150 g human diseased liver tissue derived from ALF (n = 3) and CLD (n = 6) patients. The liver tissue was enzymatically digested with collagenase type 1A (Sigma), filtered and purified further via density gradient centrifugation over Percoll (Amersham Biosciences, Little Chalfont, UK). BECs were then extracted from the mixed nonparenchymal population via magnetic selection, as described previously^27^ and were cultured for 1 week (37°C with 5% CO_2_) in rat-tail collagen-coated flasks. After the first passage, BECs were cultured for 24 hours and BEC culture supernatants were collected to soluble B7 using ELISA.

### Hepatic Endothelial Sinusoidal Cells Exposure to Plasma from ALF Patients

Forty-eight-well cell culture plate (Costar; Corning Inc) were precoated with type 1 rat-tail collagen (Sigma-Aldrich). HSECs from an explanted liver with liver cirrhosis were cultured until confluent. The cells were subsequently detached using cell-dissociating reagent TrypLE Express Enzyme (Gibco, Life Technologies) for 5 minutes at 37°C, deactivated by addition of phosphate-buffered saline. A cell pellet was obtained by centrifugation at 2000 rpm for 5 minutes. To each well was added 2 × 10^4^ HSECs in a volume of 500 μL media (human endothelial serum free media, 10% human AB serum, recombinant vascular endothelial growth factor, and hepatocyte growth factor (PeproTech EC Ltd, London, UK) both at concentrations of 10 ng/mL). After 24-hour incubation, cells were washed and cultured in serum free media supplemented with 2% fetal bovine serum and recombinant vascular endothelial growth factor and hepatocyte growth factor at 10 ng/mL (eBioscience). After 24-hour culture, cells were washed and further incubated for 24 hours in the presence of sera from patients with ALF (n = 6) or healthy controls (n = 6) at 25%. Cells were further activated using 10 ng/mL recombinant human tumor necrosis factor−α (PeproTech) and 10 ng/mL IFN gamma (PeproTech). Supernatants were then collected and analyzed for sB7 molecules.

### Enzyme-Linked Immunosorbent Assay and Meso Scale Discovery Multiplex Cytokine Detection System

Soluble CD80 and CD86 were assessed in cell culture supernatants and human sera of HC (n = 10), ALF (n = 25), ACLF (n = 20), CLD (n = 15), and sepsis (n = 20) and sequential samples (D1, n = 12; D3, n = 6; D7, n = 7; D14, n = 5) using ELISA (eBioscience and Cusabio, College Park, MD, respectively). In addition, levels of sCD86 were quantified in sera from APAP-induced liver injury model murine models using ELISA (Cusabio). Quantikine ELISA was used for IL-2 detection (R&D Systems, UK). The Human TH1/TH2 10-Plex panel (Meso Scale Discovery [MSD]) was used to assess the following cytokines: IFN gamma, IL1β, IL2, IL4, IL8, IL10, IL12p70, IL13, and tumor necrosis factor−α. ELISA microplates were read using the SoftMax Pro software (Molecular Devices). MSD plates were acquired on the Sector Imager 2400 apparatus. All ELISA and MSD experiments were performed in duplicate and according to the manufacturer's instruction. Total and caspase-cleaved cytokeratin 18 (M65 and M30, respectively) were measured in sera on admission in ALF (n = 25), ACLF (n = 20), CLD (n = 15), and nonhepatic sepsis (n = 20) using ELISA (Peviva AB, Bromma Sweden), and according to manufacturer’s instructions. Levels were assessed in supernatants from APAP untreated and treated primary human hepatocytes.

### Murine Sera Samples

All research using live animals was approved by local ethics committees (Imperial College Central Animal Welfare Ethical Review Board) and was carried out under Home Office supervision in accordance with the Animal (Scientific Procedures) Act 1986 (UK). Procedures were performed under the authority of UK project licence 70/7578. All efforts were taken to minimize animal suffering. Mice were housed in specific pathogen free conditions in individually ventilated cages (Techniplast UK Ltd, Kettering, UK). The environment was controlled with a 12-hour light/dark cycle, ambient temperature of 21°C (±2°C) and humidity of 55% (±10%). Mice had access to irradiated diet (Special Diet Services UK, Essex, UK) and water (25 ppm chlorine) ad libitum except when food was withdrawn for defined experimental purposes. Mice were fasted overnight for 12 hours before intraperitoneal dosing with 325 mg/kg APAP dissolved in saline by warming to 50°C for 45 minutes. One experimental group were culled at baseline, following the overnight fast alone. Other groups of were culled at 8 hours, 24 hours, 48 hours, and 5 days after dosing with APAP. At necropsy the liver was excised; half was place in formalin for fixation and half was snap frozen in liquid nitrogen. Formalin-fixed liver tissue was paraffin-embedded, sectioned at 10 μm thickness, and mounted onto glass slides. Sections were stained with H&E.
